# Phenomenological study of international medical graduates and the supervisory relationship in Canada

**DOI:** 10.5116/ijme.6836.cbfc

**Published:** 2025-06-20

**Authors:** Brenna Lynn, Bob Bluman, Vernon Curran

**Affiliations:** 1 Division of Continuing Professional Development, Faculty of Medicine, University of British Columbia, Vancouver, BC, Canada; 2 Division of Population Health and Applied Health Sciences, Faculty of Medicine, Memorial University of Newfoundland, St. John's, NL, Canada

**Keywords:** International medical graduate, supervision, interview

## Abstract

**Objectives:**

This study explored the nature of the supervisory relationship between international medical graduates (IMGs) on temporary practice license and their supervisors, including perceptions of the roles and expectations of IMG supervisors in Canada.

**Methods:**

A qualitative phenomenological study comprising six focus group interviews was undertaken. A purposive sample of study participants was recruited via e-mail, and twenty-one supervisors and IMGs on temporary licensure practicing in British Columbia, Canada participated. Focus groups were recorded and transcribed verbatim, and data were analyzed using thematic analysis.

**Results:**

Key themes occurring from the focus groups included the Role of Supervisor, Supervisor Interaction, Supervisor Background, Benefits of Supervision, and Supervisor vs Mentor. The supervisor’s role was perceived as necessary in supporting the IMG with adjusting, transitioning, and navigating medical practice in Canada. Access and availability of the supervisor were critical, while supervisors with IMG backgrounds were more empathetic to supervisees’ needs. Having mentors, in addition to one’s supervisor, was seen as valuable in helping with personal and professional adjustments to life in a new country and a novel medical system.

**Conclusions:**

Supervisors and IMGs felt the supervisory process was beneficial, but could be enhanced to support IMGs' transition better. Supervisor availability and contact were important to foster engagement throughout the supervisory relationship. Supervisor training was essential to prepare for the supervisory role, and combined supervision and mentorship programs were considered helpful for IMGs as they adjusted to their new practices and life in a new country.

## Introduction

Numerous countries rely on a consistent influx of international medical graduates (IMGs) or physicians trained abroad to integrate into their national healthcare systems.^1^ IMGs refer to physicians who have obtained their initial medical qualifications in countries different from the host country where they are employed.^2^ In Canada, IMGs are crucial for physician resource planning and are heavily dependent upon to address staffing gaps in rural and underserved areas. In 2022, 27% of physicians in Canada were IMGs, while physicians from South Africa, India, and the United Kingdom constitute a large proportion of Canada's internationally trained medical workforce.^3^^,^^4^ South Africa is the leading country of graduation for internationally trained family medicine physicians, while India tops the list for internationally trained specialists. About 31.1% of the physician workforce in British Columbia (BC), Canada are IMGs.^3^

For many IMGs, immigrating to a new country involves significantly adjusting to a new healthcare system. They must adapt to variations in disease patterns, technological advancements, treatment options, healthcare delivery models, culture, language, lifestyle, and gender roles.^5^^-^^8^ The dissimilarities between one’s former practice context and a new host country can challenge a successful transition. IMGs often experience feelings of alienation, frustration, and isolation when joining new medical workforces. They may also struggle with the administrative elements of a new practice and find it challenging to adapt to a healthcare culture where the doctor-patient relationship is more egalitarian.^5^^,^^7^^,^^9^ Concerns about revealing vulnerabilities and expectations regarding hierarchy can hinder IMGs’ willingness to seek assistance or voice their concerns. For some, a higher level of support and mentorship is essential for successful adaptation and adjustment.^6^^,^^10^

Training and support programs, such as orientation and communication skills courses or workshops, can help IMGs transition to practice in their new host countries.^2^^,^^9^ It is recommended that mandatory orientation occur as early as possible, before IMGs start their practice, to help them acclimate effectively to the new healthcare environment. Such orientation may include covering topics such as communication, cultural awareness, and ethical guidelines.^2^ Programming that is most effective in supporting IMG adjustment includes those that address the individual needs of IMGs and offer ongoing guidance and advisement from peers and supervisors. Programs that include ‘buddying’ or peer support and enhanced supervision can facilitate the successful transition of IMGs to their new practice and organizational contexts.^2^

The integration of IMGs into the medical workforce in Canada varies across jurisdictions depending on one’s postgraduate educational background and practice experience. In some jurisdictions, IMGs may be deemed ‘practice ready’ by a respective provincial medical regulatory authority and begin practice under certain conditions that may or may not involve the assessment of clinical skills. For these IMGs, most Canadian regulatory authorities place them on a provisional (also called temporary/restricted/conditional/special) register that allows them to practice clinically under supervision. To obtain full licensure, IMGs must pass national licensing examinations by the Medical Council of Canada, College of Family Physicians of Canada, and/or the Royal College of Physicians and Surgeons of Canada during their provisional register period.^11^

In British Columbia, IMGs can apply for licensure in the provisional, clinical observer, assessment, or associate physician classes.^12^ These licensure classes are intended for physicians or surgeons who have earned their medical degree from a medical school outside Canada recognized in the World Directory of Medical Schools. The provisional class of licensure allows a registrant to practice medicine within the boundaries defined by their education, qualifications, and experience. This licensure class requires that the health authority provide a sponsorship letter that identifies a supervisor satisfactory to the Registration Committee. Such supervisors are expected to provide supervision proportionate to the registrant’s experience and competence and submit reports regarding the registrant’s performance as directed by the College of Physicians and Surgeons of British Columbia.^13^

Physicians who supervise IMGs are crucial to their successful transition and integration into medical practice in Canada. Supervising IMGs is a significant and complex responsibility, as it can take place in diverse settings and serve multiple functions and purposes. In medicine, supervision entails the monitoring, guidance, and feedback related to personal, professional, and educational development, all within the scope of a physician’s care for patients.^14^ This involves supporting supervisees in enhancing their clinical skills, decision-making, and overall competency in delivering patient care. There is broad consensus that supervision in medicine encompasses three main functions: educational, supportive, and managerial. Additionally, it is widely agreed that the essential aspects of supervision include ensuring patient safety and promoting professional development. These functions work together to create a comprehensive approach to supporting the supervisee and the quality of patient care.^15^

An important aspect of supervision is the relationship between the supervisor and the supervisee. The quality of this relationship is a crucial determinant of the effectiveness of the supervisory process and significantly influences the satisfaction of both the supervisor and the IMG. A supportive and trusting relationship fosters open communication, feedback, and a conducive learning environment, ultimately enhancing the supervisory experience.^2^^,^^14^^,^^15^^-^^18^ However, there is minimal published work on the supervision of IMGs and the experiences of supervisors of IMGs.^15^^,^^19^ In Canada, research around the nature of the supervisory relationship involving IMGs practicing under temporary licensure is even more limited. However, the supervisory relationship and the supervisor’s role are critical elements in facilitating IMGs’ successful transition to practice within the Canadian healthcare system. A strong, supportive supervisory relationship helps IMGs navigate challenges, adapt to the new environment, and build the necessary skills and confidence for effective patient care. Supportive activities such as supervision and mentoring also offer potential retention strategies by enhancing confidence and fostering supportive workplaces that can increase professional satisfaction.^20^^,^^21^

Bordin’s^22^ ‘working alliance-based model of supervision’ is one theoretical model that emerges from the ‘therapeutic alliance’ perspective and may offer some guidance on better understanding supervision. This supervision model has three main parts: mutual agreement on goals, agreement on tasks, and a strong emotional bond between supervisor and supervisee. Together, these components contribute to a productive supervisory experience that enhances the learning and integration of IMGs into the healthcare system.^17^ An improved understanding of the supervisor’s role and the supervisory relationship in supporting the successful transition of IMGs in their new host countries can inform efforts to improve IMG supervision.^1^^,^^2^^,^^18^ However, Moran and colleague’s ^23^ integrative review of the literature found limited research surrounding supervision interventions amongst rural and remote health workforces, which often include a high proportion of IMGs.

This study explored the nature of the supervisory relationship between IMGs on temporary practice license and their supervisors, including perceptions of the roles and expectations of IMG supervisors in Canada.

## Methods

### Study Design

Phenomenology is a qualitative research methodology focusing on understanding participants’ perceptions, feelings, and experiences regarding a specific phenomenon. Phenomenological research aims to uncover that phenomenon’s ‘essential structures’ by conducting in-depth interviews with individuals who have directly experienced it. This approach seeks to capture the richness and complexity of human experiences. It allows researchers to understand how individuals make sense of their world and the meanings they attach to their experiences.^24^ Focus group interviews were conducted between July and September 2010 with a purposive sample of IMG supervisors and IMGs who had participated in a supervisory relationship as required for temporary licensure in British Columbia, Canada. A phenomenological approach was adopted to explore the nature of the supervisory relationship between IMGs and their supervisors, including perceptions and experiences of the role of supervisor of IMGs.

Descriptive phenomenology was adopted in this context to emphasize the description of participants’ experiences and identify commonalities across their perspectives.^25^ This approach allows researchers to capture the essence of participants’ experiences in detail. Additionally, several authors in health research have supported the use of the phenomenological focus group interview method.^26^^-^^28^ This method facilitates rich discussions among participants, allowing for the exploration of shared experiences and insights, which enhances the depth of understanding of the phenomenon under investigation. Focus group interviews also enable participants to engage in discussions with peers that may generate additional feedback that may not have been obtained through one-on-one interviews.

All members of the primary research team (BL, BB, VC) have held senior academic administrative positions with university offices of continuing professional development for physicians and other health professions. They have provided oversight for assessment, training, and orientation programs for IMGs and orientation workshops for IMG supervisors and/or assessors. Members of the research team also had considerable prior experience with conducting and analyzing qualitative research data. Their interests included a better understanding of enhancing professional development programming that supports the successful transition of IMGs to practice in their new host country. The investigators do not have personal or professional experiences as IMGs but utilized the ‘bracketing’ principle as Edmund Husserl developed.^29^ By employing this approach, the researchers aimed to set aside any biases and preconceptions to the greatest extent possible. This process is critical in phenomenological research, as it allows researchers to approach the study participants’ experiences and perceptions from a fresh and open perspective, thereby enhancing the validity and depth of the findings. By minimizing the influence of their own experiences, the investigators sought to authentically capture and understand the lived experiences of the IMGs in the study. The team discussed the final coding analysis and thematic categories to minimize misconceptions or biases in interpretation.

### Participant recruitment and sampling

A purposive sample of study participants was recruited via e-mail invitations sent to IMG supervisors and IMGs registered with the College of Physicians and Surgeons of British Columbia and past participants in the British Columbia Physician Integration Program (BC-PIP) coordinated through the University of British Columbia Division of Continuing Professional Development. The purpose and rationale for the study were communicated in both the invitation message and the ethical consent form that study participants were required to sign.

### Consent and data collection

Ethics approval was received from the University of British Columbia Behavioral Research Ethics Board, #H09-01531. We obtained informed consent with a consent form for participation in this study. Focus group interviews were 60-90 minutes long, co-facilitated by a project manager and research assistant, and conducted by audio-teleconferencing. The BC-PIP medical director and a primary research team (BL) member attended and observed the focus group interviews but were non-participants.

Focus group interview scripts were shared with participants in advance. They consisted of 12 questions for supervisors and 11 for IMGs. They encompassed questions around general perspectives on the nature of IMG supervision, supervisor roles and responsibilities, attitudes towards supervision and mentorship, and perceived factors impacting professional relationships between supervisors and IMGs (Appendix). Drafts of the focus group interview script were reviewed by a project advisory committee comprising members of the primary research team (BL, BB), the program medical director, and physician representatives. Key informant interviews with physician leaders were also conducted and informed the questions asked in the focus group interviews.

### Qualitative data analysis

Focus groups were recorded and transcribed verbatim, continuing until data saturation was achieved, which indicates that no new information or themes emerged from the discussions. The data were analyzed using a thematic analysis technique to organize, identify, and document patterns or themes within the dataset.^29^ The analysis process involved multiple steps: transcripts were independently reviewed by members of the research team (VC, BL, BB), who convened to discuss the identified themes and generate an initial list of codes. This collaborative approach helped ensure consistency and depth in understanding the data. Following this, the transcripts underwent independent first-level coding, where initial codes were assigned to text segments. The second-level coding involved a comparative analysis of these codes. It allowed the researchers to contrast them and form coherent thematic categories and sub-categories that encapsulated the participants’ experiences and perspectives. This rigorous analytical process ensures a comprehensive understanding of the phenomena explored in the study. A copy of the final report summarizing key findings from the focus group sessions was distributed to study participants.

## Results

Twenty-nine (n=29) supervisors were interested in participating in this study, with nine (n=9) attending one of three focus groups. Of the 289 IMGs invited to participate, 12 attended another set of three focus groups. Table 1 summarizes the focus group respondents' background characteristics. The supervisors included four family physicians (FPs) and five specialists whose medical practices were either private (n=4) or group (n=5; typically, hospital) settings practicing across five of the six health authorities in British Columbia. Four supervisors were IMGs, while 5 were Canadian medical graduates (CMGs). The IMG respondents included 7 FPs and five specialists who started practicing in BC between 2007 and 2010 in private or group practice settings in various health authorities in BC. Some IMGs worked in the same practice setting as their supervisor (within the same community), while other IMGs worked in different communities from their supervisor. Before coming to Canada, the IMGs had practiced medicine for 5 to 20 years in countries such as the United Kingdom, India, South Africa, and the United States.

**Figure 1:**
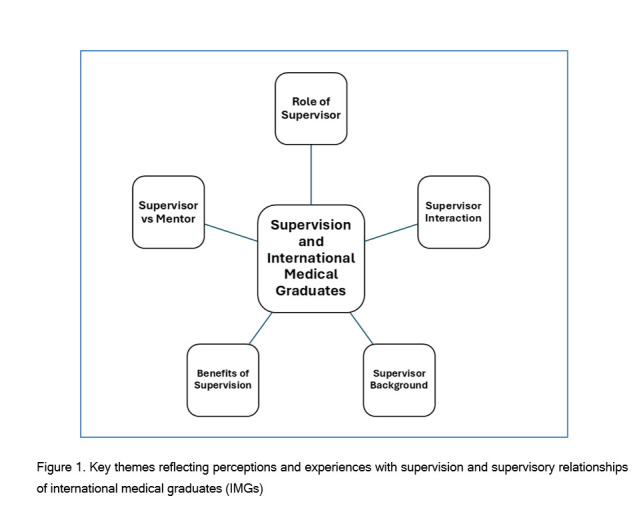
Key themes reflecting perceptions and experiences with supervision and supervisory relationships of international medical graduates (IMGs)

Several key themes occurred from the analyses of the focus groups with supervisors and IMG respondents (Figure 1), including the Role of Supervisor, Supervisor Interaction, Supervisor Background, Benefits of Supervision, and Supervisor vs Mentor.

### Role of Supervisor

Supervisors and IMGs described their perceptions of and experiences with the supervisory relationship, with a critical focus on the supervisor's ‘role’ in providing supervision during the IMG’s temporary licensure term. Several sub-themes were created around this role of the supervisor.

#### Guide to the local setting and healthcare system

Supervisors described how they perceived their role as a “guide” to IMGs, assisting them with their “adaptation” and “integration” into medical practice in Canada and helping them understand the way things worked in the local practice setting and the health system itself. This included guidance around making referrals and, in some instances, offering advice on patient care:

“…it was more to get him used to the systems, you know, that pertain to Canada, both Canada and BC in particular. And the local, the local environment. So it’s really teaching the tricks of the trade as it were, drugs, sources of referral, kinds of practice, that sort of thing, rather than medicine itself.” (Supervisor 20, Male, Urban, Family Practice)

IMGs also echoed the important role of the supervisor in helping the IMG physician become familiar with the Canadian and local healthcare system and the way things worked:

“I don’t know, my supervisor, because we had electronic medical records, she read everything that I wrote and whenever she had any concerns or anything regarding my performance, I went to her house and we discussed what was missing and things like that. So I think it’s very much needed that at the beginning of the practice…. the reality in Canada is very different from any other part of the world. The kind of medicine we practice here is different from any part of the world so it’s very important that you have what you do checked and, um, so that you can learn, right, so that’s the idea…” (IMG 12, Female, Urban, Family Practice)

**Table 1:** Focus Group Respondents’ Characteristics

Respondent	Role	Gender	Geographyof practicesetting	Area of medicalpractice
1	Supervisor	Female	Urban	Family Practice
2	Supervisor	Male	Urban	Specialist Psychiatry
3	IMG	Male	Rural	Specialist Internal Med
4	IMG	Female	Urban	Specialist Psychiatry
5	Supervisor	Male	Urban	Specialist Psychiatry
6	Supervisor	Female	Urban	Specialist Neonatologist
7	IMG	Male	Urban	Family Practice
8	IMG	Female	Rural	Family Practice
9	IMG	Female	Urban	Specialist OBGyn
10	IMG	Male	Rural	Family Practice
11	IMG	Male	Rural	Family Practice
12	IMG	Female	Urban	Family Practice
13	Supervisor	Male	Rural	Family Practice
14	IMG	Male	Urban	Specialist Pediatrics
15	IMG	Female	Urban	Specialist Psychiatry
16	IMG	Male	Urban	Family Practice
17	IMG	Male	Urban	Family Practice
18	Supervisor	Male	Urban	Specialist Psychiatry
19	Supervisor	Male	Urban	Specialist Internal Med
20	Supervisor	Male	Urban	Family Practice
21	Supervisor	Male	Urban	Family Practice

#### Understanding needs of the IMG

Some supervisors also described the need to take time to explore the needs of the IMG based on their past training and practice experience. It was felt the level of supervision or guidance they offered would be dependent on the IMG needs, and the level of guidance could taper off over time as the individual became more comfortable:

“We spend significant time at the beginning to explore what the people come to us, um, in what degree of the training that they had in the past and depend on that, …a lot of time needs to be spent on talking with people about policy, about accepting health care, about our health care system, how is it different than others so that you know they can learn right from the beginning to avoid certain mistakes administratively or medical/legal issues.” (Supervisor 2, Male, Urban, Specialist, Psychiatry)

“I think as the process progresses and the IMG has been with you for a longer period of time, obviously I think your role changes and it becomes more just sort of watching and observing what they’re doing rather than you know being involved over time obviously.” (Supervisor 1, Female, Urban, Family Practice)

#### Challenges with access to and regular contact with supervisor

However, some IMGs did not feel the supervisor's role was particularly clear or well understood, and for some, accessibility to their supervisor or even contact with the supervisor was lacking. There was a belief that there needed to be a more formalized structure to the supervisory relationship and the role of the supervisor:

“To be honest I didn’t know what to expect because I didn’t really know what the role of a medical supervisor actually is, you know.” (IMG 7, Male, Urban, Family Practice)

“I think that some work could be done to formalize that process without it being, um, making it a rather tedious and painful process…consider having formal parts of the process such as doing case reviews together, discussing cases, um, independently and talk about management and difficulties or discussing difficult cases and those being referred to where needed. I think it lacks a lot of structure….” (IMG 17, Male, Urban, Family Practice)

#### Team Supervision

The important role of other practice team members in providing guidance and advice to the IMGs was also recognized and discussed by both supervisors and IMGs. It was recognized that others in the practice setting also played key supportive roles for the IMG during the supervisory period as well.

“…start at obviously asking a lot more questions like we all did and but asking those questions as time go on and it's not particularly me who come will running to for support or backup as who will be, actually who's ever close and whosever in the building” (Supervisor 13, Male, Rural, Family Practice)

“…as a group supervisor I'm really not the person who's normally down supervising but we do it all as a group.” (Supervisor 13, Male, Rural, Family Practice)

### Supervisor Interaction

Supervisors and IMGs described the nature and characteristics of the supervisory relationships they experienced, including the level and type of interactions that occurred. IMGs reported challenges with access to their supervisors in many instances and even minimal to no contact with their supervisors during the supervisory period. Some supervisors reported they had consistent connections with their supervisees, whether in-person, by e-mail, or by telephone. However, some supervisors did recognize that more direct, in-person, and on-site supervision may have been preferred:

“So after I’d moved I was still the supervisor and I would still you know be available for questions and that kind of thing, but I wasn’t there on a daily basis to supervise. And, um, it was just a very dissatisfying experience for myself as well as the IMG because I think the role of a supervisor is to actually be there, um, on a, you know, fairly continuous basis to supervise and answer questions and give advice and it’s a lot more difficult over the phone than it is in practice.” (Supervisor 6, Female, Urban, Specialist Neonatologist)

Some IMGs described a general lack of contact with their supervisors altogether, and some discussed their perceptions that the supervisor did not really have a good understanding of the background of the supervisee before they arrived, nor seemed very interested in acting as a supervisor:

“Actually for me if I’m supposed to have the meeting on a regular basis but my experience in BC I never had any actual meeting with my supervision. So I just got some advice indirectly or by phone any time it’s needed or something happened. I never had any direct supervision with the patient care…I never had an actual meeting with my supervisor.” (IMG 9, Female, Urban, Specialist OBGyn)

“I just have a little bit of concern about how little knowledge the supervisors have in advance of the mentee arriving and I think that that is something that could be easily addressed without it being too formal or very long winded.” (IMG 17, Male, Urban, Family Practice)

There were mixed perceptions of the level of formality of the interactions expected by the IMGs. Some felt that more regular contact and interaction would have been preferred, while others felt that a less “informal relationship” worked better:

“So in my case I quite enjoy the informal relationship. It doesn’t feel like my supervisor is superior to me or it’s just a very casual, I’m able to ask questions and they ask questions from me and, um, yeah I quite appreciate that.” (IMG 10, Male, Rural Family Practice)

Some IMGs felt the supervisor could have offered more advice or assistance in preparing for the national exams to achieve full licensure:

“I never had any help from the supervisors regarding the exams. I knew there were exams, I knew lots of other IMG’s and I knew IMG’s who have already passed the exam and they had materials for them so I just prepared myself. I didn’t count on any help from the supervisor. This is something that most IMG’s do by themselves.” (IMG 12, Female, Urban, Family Practice)

### Supervisor Background

The supervisor's background characteristics, whether the person should be a Canadian medical graduate or an IMG, were also discussed, and both supervisors and IMGs shared opinions. Generally, most IMGs felt that having a supervisor with an IMG background would be helpful, as the person may be more empathetic and better understand the IMG's past medical experience and training, which could be useful in offering guidance to the IMG. However, others felt that having a Canadian medical graduate as a supervisor would also provide benefits.

“Well I was previously an IMG myself and I actually think, um, in my opinion you understand the foreign graduate a little bit better and especially if the culture is the same and you come from the same country it’s a lot easier to guide them, not only in integrating into the medical system, but also integrating in the Canadian culture and everyday life.” (Supervisor 1, Female, Urban, Family Practice)

### Benefits of Supervision

Despite some challenges reported by some IMG respondents regarding their experiences with the supervisory relationship and a lack of connection with their supervisor, many IMGs and supervisors expressed satisfaction with the supervisory process and experience. They described the benefits of having supervision during the temporary license period.

“I’d also say there’s a big benefit through the communities that we work in, um, for us to be doing supervision so that we can integrate these physicians into the Canadian culture and, um, have them practice medicine…” (Supervisor 1, Female, Urban, Family Practice)

“…just having the supervisor is positive because you are, you feel more comfortable in the back of your mind and you’re feeling anytime, any place, any circumstance, so that somebody is there if you have any questions they can answer. This is a good feeling actually, this is good experience for me.” (IMG 9, Female, Urban, Specialist OBGyn)

### Supervision and Mentorship

Supervisors and IMGs were also asked to discuss their perceptions of the supervisor’s role regarding offering ‘mentorship’ and supervision. There were mixed observations and perceptions regarding the role of the supervisor in offering mentorship. Some felt that the supervisor was also providing mentorship, while others thought that the roles of supervisor and mentor should be separate:

“If we go for a broad definition of mentoring, mentoring is like being a teacher, a professor that doesn’t only help you from the academic point of view, but helps you also like to kind of overcome those difficulties that my colleagues have said at the beginning. Like if you are from a different country,

the cultural things, I remember that it was a personal experience...So this to me is mentoring. Like explaining me in a way how is the culture of the place. He also helped me to find a place to rent, um, taught me to go to Ikea to buy furniture. So this to me is mentoring...mentoring for me is something more broader. It’s a little bit of a touch of friendship, a touch of friendship and help yourself go through all those, um, enormous difficulties that all of us who are IMG’s and succeed here in Canada have to go through.” (IMG 12, Female, Urban, Family Practice)

“Well I think that it should be one and I’ll tell you why, because, um, I think that, um, how is a supervisor going to know whether you are practicing up to standard without looking punitive and critical and basically to get to know you is to actually mentor you, to create an environment where you feel safe to ask questions and you feel safe to ask advice and, um, to complain maybe or to talk about the difficulties you are having. I think it should be one person because that will make the whole supervision process a lot friendlier and a lot more acceptable. That’s just my opinion.” (IMG 15, Female, Urban, Specialist Psychiatry)

## Discussion

The findings from the focus group interviews raise several important implications for enhancing policy and programming surrounding the supervision of provisionally licensed IMGs and improving support for supervisors and IMGs to enhance the quality of such supervisory relationships. A number of authors have identified a lack of commitment, time, interest, contact, or availability of the supervisor as critical deterrents to the quality of the supervisory relationship.^14^^-^^17^ Similarly, in our study, several IMGs reported challenges with access to their supervisors, minimal to no contact in some instances, perceptions that supervisors did not really know the background of the IMG, and disinterest in acting as supervisors in some instances. Even though the IMGs being supervised were provisionally licensed and not postgraduate trainees, the level of accessibility and opportunity for regular meetings with one’s supervisee does offer an important opportunity to identify potential problems and is a critical element of an effective supervisory relationship.^15^^,^^17^ Strategies around co-location, better remuneration of supervisors, and protected time to increase supervisor availability and ensure more regular formal contact between IMGs and their supervisors should be considered to facilitate greater engagement and feedback throughout the supervisory relationship.

Supervisors and IMGs did believe the supervisory process was beneficial and, with improvements, could be of even greater support to IMGs as they transitioned into a new healthcare system and workplace environments. Ongoing support is essential for IMGs during their transition, as it plays a significant role in helping them manage stress and adjust healthily. Research indicates that high levels of support at an early stage are particularly beneficial, as they can aid in identifying initial problems and preventing their escalation.^2^ This proactive approach fosters a smoother transition into their medical practice and contributes to the overall well-being and resilience of IMGs as they navigate the challenges associated with adapting to a new healthcare environment and culture. By ensuring that IMGs receive timely and adequate support, healthcare systems can enhance the integration and satisfaction of these professionals in their roles.

Individual needs assessment has been identified as an important aspect of effective supervision as well in order to tailor the nature of supervision and level of support required by the IMG.^2^ When working with individual IMGs, it is essential to consider their past experiences within different healthcare systems and the social and cultural contexts they have come from. This understanding can significantly inform the level of supervision required and identify areas for further and ongoing support.^1^ Recognizing these diverse backgrounds allows for a more tailored approach to supervision and support, ensuring that IMGs receive the guidance they need to effectively navigate their new environments. By acknowledging the unique challenges and skills that each IMG brings, healthcare organizations can better facilitate their integration and professional development, ultimately enhancing their performance and job satisfaction in the new context. Some supervisors described the need to take time to explore the IMG's needs based on their past training and practice experience. This was believed to be useful in initiating the relationship and identifying the goals for the supervisory process. This finding has important implications for enhancing supervisor training and/or orientation to equip supervisors with knowledge and techniques for developing supervisory goals for IMGs and ensuring appropriate and relevant supports are in place throughout the supervisory process.

There were mixed perceptions around whether the supervisor should have an IMG background or be a Canadian medical graduate, with pros and cons to either. Most IMGs felt that having a supervisor with an IMG background might enable that person to demonstrate greater empathy and understanding of the IMG’s needs. Implementing a buddying or mentor arrangement may also offer a supplementary means to provide ongoing support within and outside the workplace. According to Kehoe and colleagues,^2^ understanding the individual needs of IMGs can help determine whether they would gain more from a host-country buddy, an IMG buddy, or potentially both. Having a personal buddy who can offer information and support as needed can significantly reduce the stress and anxiety often experienced during the transition process. Buddies who are IMGs themselves possess valuable insider knowledge about the unique challenges that lie ahead, enabling them to offer relatable guidance and support. Additionally, observing how other IMGs have successfully navigated their own circumstances can enhance self-efficacy, making new arrivals feel more confident in their ability to adapt to their new work environment. This peer support system fosters a sense of belonging and facilitates better overall work adjustment, ultimately contributing to the success and well-being of IMGs in their new roles.^2^ Dual but separate supervision and mentorship programs may provide IMGs with the different types of support they require as they transition to a new healthcare system and adjust personally to living in a new country.

Both supervisors and IMGs recognized and discussed the important role of other practice team members in providing guidance and advice to IMGs. Peer support in practice is essential, and continuing support from all interdisciplinary team members is crucial during the period of transition and integration into the workplace.^6^ This team effort can provide additional clinical and educational input, essential in helping IMGs navigate their new roles effectively. Furthermore, team members are often well-positioned to identify challenges the supervisee may encounter, allowing timely interventions to address these issues.^17^ By collaborating and sharing responsibilities, the wider practice team can create a more comprehensive support network for IMGs, ensuring they receive the guidance and resources necessary to enhance their professional development and integration into the healthcare setting. This collaborative approach benefits the IMGs and strengthens the healthcare team's overall functionality, fostering an environment conducive to learning and growth.

Several authors have also identified the important need for ongoing support and training for IMG supervisors to effectively facilitate the peer relationship inherent in IMG supervision.^1^^,^^6^^,^^15^^,^^17^ Formalized training and orientation activities for supervisors are necessary to introduce them to the purpose of the supervisory process and relationship, the role of the supervisor and expectations, and useful techniques or tools to enhance the nature and level of supervision provided. Jackson and colleagues^17^ found that providing peer support for supervisors, such as through supervisors’ workshops, is beneficial in preparing and developing supervisors for supporting IMGs. These workshops offer a platform for supervisors to share experiences, discuss challenges, and exchange strategies for effective supervision. Such training may encompass learning about the ‘medical culture’ of the IMGs’ countries of origin and cultural sensitivity training to allow IMGs and supervisors to discuss cultural differences.^7^ In the Canadian Task Force Report on the Licensure of International Medical Graduates, the Federal/Provincial/Territorial Advisory Committee on Health Delivery and Human Resources^30^ made several recommendations to enhance the support provided to faculty and physicians who work with IMGs. One key recommendation was the development of comprehensive orientation programs designed to better equip these stakeholders with the skills and knowledge to effectively mentor and guide IMGs in their clinical roles. In 2006, the Association of Faculties of Medicine of Canada established a faculty development program for teachers of IMGs.^31^ The Division of Continuing Professional Development with the University of British Columbia, Faculty of Medicine, has offered one-day faculty development workshops for supervisors of provisionally licensed IMGs. The purpose of these workshops was to assist supervisors in diverse settings to work with IMGs in an effective and collaborative manner to enhance their learning and practice experiences. Topics covered included expectations of supervisors of temporarily licensed IMGs, understanding the IMGs’ world, promoting patient-centered care and effective communication, assessing learning needs, providing effective feedback, and ethical and legal issues related to supervision.

### Limitations

Although the qualitative data reported in this paper are from some time ago, the findings are very much relevant and valuable for several reasons. Firstly, the focus group methodology used to collect the original data for this study was conducted in a highly rigorous manner. The study results are still applicable today and serve as a reliable reference point for any further investigations that may occur around supervision of IMGs. Given the lack of literature and research of IMG supervision in particular, the study findings offer important foundational insights that set the context for present-day trends in this field. Furthermore, historical data is crucial for longitudinal studies because it enables comparisons over time and reveals how perspectives or critical insights about the evolution of IMG supervision may have changed. Although the focus groups yielded sufficient data to achieve saturation and identify key themes, physicians no longer currently supervising an IMG during the study were excluded. To further our understanding, a broader sampling might have resulted in a more diverse sample of supervisor respondents and provided additional information regarding supervision in BC.

## Conclusions

A large group of IMGs practice across Canada on a provisional register that encompasses the use of ‘supervision’ by a fully licensed physician for some period. According to Triscott and colleagues,^8^ IMGs bring multiple strengths to healthcare delivery in Canada, including experience and clinical expertise, high levels of education and training, the richness of diverse cultural perspectives, and positive personal attributes. Supervision is a critical component of medical education at all levels, ensuring trainees receive the guidance and support necessary to develop their clinical skills and knowledge. However, research specifically focused on supervising IMGs operating under provisional licensure circumstances has been limited. Supervising practicing IMGs is a distinctive area in medical supervision and thus requires a specific understanding of the context and dynamics of IMG supervision. In particular, specific considerations are needed for regulators and medical educators. Needs-based faculty development programming is required to adequately support supervisors of practicing IMGs so that they can fulfill their role. In order to successfully integrate into the health system, IMGs may benefit from an identified mentor in addition to the supervisor so that there is safe (non-reporting) support available to the IMGs. The qualitative data analyzed and reported in this paper addresses a unique gap in our knowledge around some of the key factors that support the transition of IMGs to practice. The findings offer further insight into ways to enhance the supervision of IMGs and improve the supervisory process, which in turn may improve IMG adjustment and transition and foster better retention of IMGs in their new practice settings.

### Availability of data and materials

The datasets used and/or analysed during the current study are available from the corresponding author on reasonable request.

### Acknowledgements

The authors would like to acknowledge the contributions of Tunde Olatunbosun, Dr. Harry Karlinsky, and Chloe Burnett.

This work was supported with funding from the Canadian Medical Protective Agency collaborative research and risk management program development grant.

### Conflict of Interest

The author declares that there is no conflict of interest.

## Supplementary material

Supplementary file 1 Appendix. Focus Group Questions
