# Supplementary material for: Phenomenological study of international medical graduates and the supervisory relationship in Canada
Source: Int J Med Educ. 2025 Jun 20;16:107–17. doi: 10.5116/ijme.6836.cbfc (PMC12701529; doi:10.5116/ijme.6836.cbfc)
Supplement: Supplementary file 1 — Appendix. Focus Group Questions [file ijme-16-107-S1.pdf]

Appendix

IMG Focus Group Questions

1. What are your thoughts on the supervision and supervisory relationship if your supervising physician was previously an IMG? (*Prompt*: Is it important/beneficial or not?)
2. From your experience, does the supervision occur in a meeting format or is it during direct patient contact?
3. What are your thoughts on providing feedback or evaluation about your supervising physician?
   Probe: To whom should feedback and evaluation be sent to (*Prompt*: the College, sponsor, or supervisor)?
4. From your perspective, what, if any, are the main positive experiences from the supervision experience between you and your supervisor?
5. From your perspective, what, if any, are the main challenges from the supervision experience between you and your supervisor?
   Probe: How could these challenges be addressed?
6. If applicable, how would you approach a situation where you experience serious difficulty with your supervising physician?
   (Prompt: difference of opinion, lack of supervision/availability, being over worked, etc)
7. Are there situations, hypothetical or otherwise, where you feel the professional relationship between you and your supervisor might become strained?
   (Prompt: supervisor is department head, sharing financial relationship within a practice setting, etc)
8. What are your thoughts on having both a supervising physician and a separate identified mentor for an IMG's practice on the provisional register?
   Prompt: The supervisor's role is to ensure the IMG practices to standard of care expected; the mentor's role is to be available to assist the IMG as requested.
9. We have circulated a CPSBC document called "Expectations for Supervising College Members". It suggests a few duties of the supervisors when they supervise provincially licensed colleagues. Have you seen it before? Are you familiar with this document?

   According to your experience, do you have thoughts on each of these supervisory responsibilities?
   - Being available to provide advice and support to the IMGs
   - Assisting the IMGs in meeting the terms of their provisional registration, such as advising the IMG how to prepare for the exams
   - Being familiar with IMG's pattern of practice and quality of care
   - Reporting to the College and the sponsor
10. Do you have concern about the amount of clinical workload your supervisor allocated to you? Please explain.
11. In closing, do you have any comments in regard to the supervision process of IMGs in BC that we have not already discussed?

Supervising Physicians Focus Group Questions

1. Do you practice in the same location as your IMG? (e.g. clinic, department, hospital)
2. In the College's Expectation of Supervising College Members, it is stated that the degree of supervision depends on the level of experience and competency of the supervisee. In your experience, did the nature of your supervision on the IMG change over the time? Please explain?
3. What are your thoughts on the impact to the supervisory role if a supervising physician was previously an IMG?
4. What are your thoughts on supervising more than one IMG at a time? *Prompt*: Can it impact a supervisor's ability to provide adequate support for their IMG(s).
5. Have you received feedback and/or an evaluation on your supervision of IMGs?
   a. If yes, please explain? (*Prompt:* from who)

b. If no, what are your thoughts on receiving such feedback and/or evaluation?

Probe: Do you have suggestions on from whom and how the feedback and/or evaluation could be structured? Please describe. (*Prompt*: from IMGs, sponsors, the College, etc)

6. From your perspective, what, if any, are the main positive experiences from IMG supervision? Please describe.

7. The College suggested a few duties for the supervisors in the Expectation of Supervising College Members. If applicable, do you have thoughts on any potential dilemmas or challenges surrounding these supervisory responsibilities when supervising IMGs? For examples,
   - Being available to provide advice and support to the IMGs
   - Assisting the IMGs in meeting the terms of their provisional registration, such as advising the IMG how to prepare for the exams
   - Being familiar with IMG's pattern of practice and quality of care
   - Reporting to the College and the sponsor

   Probe: What do you think the major factors are in the potential dilemmas or challenges? (*Prompt*: personal, interpersonal, patient, institutional, etc)

8. When reporting to the College or to an IMG's sponsor, are there considerations that might affect the tone (*Prompt*: less negative) of your reporting? (*Prompt*: impacts on your IMG, their community, your practice setting, implications of withdrawing supervision)

9. During the last year's UBC CPD faculty development workshop for IMG supervisors, some supervisors said, 'my IMG has more specialized knowledge than what I have'. What are your thoughts on supervision in such situation?

   Prompt: Has your IMG come to you with a question or request that you did not feel equipped to answer?

   If yes: How was the situation handled?

   If no: How would you handle the situation?

   Probe: In regard to monitoring and assessing areas such as your IMG's quality of care or professional attitude and interaction, were there ever situations where you feel unable to provide adequate feedback?

10. What are your thoughts on IMGs having both a supervising physician and a separate identified mentor for their practice on the provisional register?

    (Prompt: The supervisor's role is to ensure the IMG practices to standard of care expected; the mentor's role is to be available to assist the IMG as requested.)

11. Are there situations, hypothetical or otherwise, where you feel the professional relationship of the supervisor and IMG might become strained? *Prompt*: supervisor is department head; sharing financial relationship within a practice setting

12. In closing, do you have any comments in regard to your supervision on your IMG that we did not discuss?
